# Development and Validation of a Prediction Model for Severe Male Pattern Baldness in Turkish Male Population Aged 50 Years and Older

**DOI:** 10.3390/genes17101200

**Published:** 2026-09-28

**Authors:** Melek Şenışık, Gokhan Ersoy, Hamdi Ozkara, Ozlem Bulbul Ercan, Gonul Filoglu

**Affiliations:** 1 Department of Medical Services and Techniques, Plato Vocational School, Istanbul Topkapi University, 34020 Istanbul, Türkiye; 2 Department of Science, Institute of Forensic Sciences and Legal Medicine, Istanbul University-Cerrahpaşa, 34500 Istanbul, Türkiye; gonulfiloglu@yahoo.com.tr; 3 Department of Medical Sciences, Institute of Forensic Sciences and Legal Medicine, Istanbul University-Cerrahpaşa, 34500 Istanbul, Türkiye; drgokhanersoy@gmail.com; 4 Department of Urology, Cerrahpaşa Faculty of Medicine, Istanbul University-Cerrahpaşa, 34098 Istanbul, Türkiye; hozkara@iuc.edu.tr; 5 Institute of Molecular Biosciences, University of Graz, Humboldtstraße 50/EG, 8010 Graz, Austria

**Keywords:** forensic DNA phenotyping, androgenetic alopecia, male pattern baldness, single nucleotide polymorphism, SNaPshot, prediction model

## Abstract

Background/Objectives: Male pattern baldness (MPB) is a complex polygenic trait influenced by genetic and non-genetic factors, with susceptibility loci on both autosomal and X chromosomes. This study aimed to develop an exploratory genetic model for distinguishing no baldness (Hamilton–Norwood [HN] grade 1) from severe baldness (HN grades 5–7) in Turkish men aged 50 years and older. Methods: Buccal-swab samples from 144 unrelated male volunteers (30 HN grade 1 and 114 HN grades 5–7) were genotyped using a multiplex SNaPshot assay containing 23 SNPs selected from previously reported MPB-associated loci. Participants were divided into a training group (*n* = 86; 18 HN grade 1 and 68 HN grades 5–7) and a held-out test group derived from the same cohort (*n* = 58; 12 HN grade 1 and 46 HN grades 5–7). Binary logistic regression was used for model development. The held-out test group was not used for SNP selection or model choice. Discrimination was assessed using receiver operating characteristic analysis and fivefold cross-validation; calibration of the final model was additionally evaluated using the Brier score and calibration intercept and slope. Results: The final 9-SNP model yielded AUCs of 0.879 and 0.777 in the training and held-out test groups, respectively. Fivefold cross-validation produced an overall AUC of 0.786 (bootstrap 95% CI, 0.696–0.868). At the conventional threshold of 0.50, test sensitivity and specificity were 0.935 and 0.250, whereas the optimized threshold of 0.843 yielded 0.717 sensitivity and 0.750 specificity. In the held-out test group, the Brier score was 0.159, the calibration intercept was 0.568, and the calibration slope was 0.492, indicating residual overfitting despite close agreement between mean predicted and observed event frequency. Among the retained markers, rs7349332 in *WNT10A* was the only statistically significant predictor in the multivariable model. Conclusions: The 9-SNP model showed moderate discriminatory performance for severe MPB in this Turkish cohort but should be considered a candidate, proof-of-concept model. The limited and imbalanced sample, extreme-phenotype design, internal rather than external validation, and exclusion of intermediate HN grades restrict generalizability; external validation in larger independent cohorts is required before forensic implementation.

## 1. Introduction

In forensic sciences, genetic analysis of biological evidence, such as bloodstains, semen, and saliva collected from crime scenes, is crucial for establishing links between the crime, the suspect, and the victims. Forensic DNA analyses serve various purposes, including suspect identification in investigations, exoneration of the wrongfully convicted, victim identification in mass disasters, and paternity determination [1,2,3,4,5,6]. Traditionally, matching Short Tandem Repeat (STR) profiles with reference samples from known individuals is considered the gold standard in forensic identification [7,8,9]. However, in countries lacking a national DNA database, the absence of a suspect can halt investigations when using STR profiling alone, often leaving cases unresolved. In such scenarios, it is vital to extract maximum information from the available evidence. Recent advancements have shown that Single Nucleotide Polymorphism (SNP) markers significantly enhance identification capabilities [10,11,12]. SNPs are now widely used in forensic science for identification, phenotyping, and determining ancestry and lineage [12,13,14,15]. 

Phenotype-informative SNPs (PISNPs) help deduce externally visible characteristics such as hair, eye, skin colour, face shape, height, and baldness from DNA samples. These developments in Forensic DNA Phenotyping (FDP), also known as molecular eyewitness techniques, introduce innovative approaches to forensic identification, helping solve more cases [16,17,18,19,20]. FDP can be used to narrow down the suspect pool in cases with many potential suspects, offering significant advantages in terms of time, cost, and labour. Initiated in the early 2000s, FDP research initially focused on pigmentation traits such as eye, hair, and skin colour, which have well-understood genetic foundations [21,22,23,24,25]. Since then, numerous forensic analysis and prediction methods have been developed. Ongoing research has expanded FDP beyond traditional pigmentation prediction to encompass a broader range of phenotypic traits, including facial features, height, hair morphology, male pattern baldness (MPB), biogeographical ancestry, and age; at the same time, massively parallel sequencing technologies have increased the number of phenotype- and ancestry-informative markers that can be analyzed simultaneously [26,27,28,29,30,31,32,33,34]. 

MPB, also known as androgenetic alopecia (AGA), is characterized by age-related hair loss in the frontotemporal and vertex regions of the scalp, with relative preservation of hair in the occipitoparietal region, and is strongly influenced by genetic factors [31,32,35,36,37,38]. MPB follows a polygenic inheritance pattern with susceptibility loci on both the X chromosome and autosomes [39,40,41,42]. Genomic studies have identified multiple MPB-associated regions, including *AR*/*EDA2R*, *TARDBP*, *PAX1*/*FOXA2*, *WNT10A*, *SSPN*-*ITPR2*, *SETBP1*, *HDAC4*, and *HDAC9* [31,32,35,36,39,43,44]. These loci participate in biological processes relevant to androgen signaling, hair follicle cycling, and morphogenesis [45]. The strongest association has consistently been observed in the *AR*/*EDA2R* region on Xq12 [46,47,48]. On autosomes, the 20p11 locus (*PAX1*–*FOXA2*) is a major susceptibility region, with additional loci identified on chromosomes 1, 2, 3, 5, 6, 7, 12, 17, and 18, including *HDAC4*, *HDAC9*, *TARDBP*, *AUTS2*, and *SETBP1* [49,50,51]. *HDAC4* and *HDAC9* encode histone deacetylases that regulate gene expression through chromatin remodeling and have also been implicated in androgen receptor signaling [46]. Additional MPB-associated loci include *WNT10A*, *SUCNR1*-*MBNL1*, *EBF1*, and *SSPN*, with *WNT10A* showing functional involvement in the anagen phase of hair growth [35,36,39,51,52]. Reduced *WNT10A* expression has been proposed to delay the transition from telogen to anagen and shorten the anagen phase. Large-scale GWAS conducted in 2017–2018 identified more than 250 MPB-associated loci and highlighted pathways involving WNT signaling, apoptosis, androgen receptor signaling, and TGF-β [43,53,54,55]. Functional studies have further demonstrated altered *EDA2R* expression in balding dermal papilla cells and an interaction between *EBF1* and *WNT10A* in hair follicle cycling [36,56]. Collectively, these findings support a highly polygenic architecture involving multiple functionally relevant loci.

Several SNP-based prediction models for MPB have been developed. Marcińska et al. [39] analyzed 50 MPB-associated SNPs in European men and reported AUC values of 0.762 and 0.864 for 5-SNP and 20-SNP models, respectively. Liu et al. [52] reported an AUC of approximately 0.74 using a 14-SNP model, whereas Hagenaars et al. [53] achieved an AUC of approximately 0.78 for severe hair loss in a large UK Biobank cohort Chen et al. [57] subsequently evaluated larger SNP-based models in independent European datasets. These studies demonstrate the potential of genetic markers for MPB prediction while also showing that predictive performance depends on phenotype definition, marker composition, age, and population background.

This study aimed to develop an MPB prediction model using nine phenotype-informative SNP (PISNP) markers from seven MPB-associated loci—*AR*/*EDA2R*, *TARDBP*, *WNT10A*, *HDAC4*, *HDAC9*, *SSPN*-*ITPR2*, and *SETBP1*—selected after evaluation of 23 candidate PISNP markers from 12 loci. The 23 candidate markers were selected according to predefined criteria, including replication in large-scale GWAS and meta-analyses of MPB (particularly the *AR*/*EDA2R* and 20p11/*PAX1*–*FOXA2* regions), proximity to genes associated with hair follicle biology, androgen signaling, and WNT/apoptosis pathways, appropriate minor allele frequencies in European and Turkish populations, low linkage disequilibrium (LD) to maximize independent information, and technical suitability for forensic applications. Priority was also given to loci previously included in predictive models, particularly *AR*/*EDA2R*, 20p11/*PAX1*–*FOXA2*, and *WNT10A*. Genotype profiles were analyzed in 144 Turkish male volunteers aged 50 years and older, classified as HN grade 1 (no baldness) or HN grades 5–7 (severe baldness). The study was designed to evaluate the discriminatory potential of the selected markers for severe MPB and their possible contribution to forensic DNA phenotyping.

## 2. Materials and Methods

### 2.1. Sample Collection, DNA Extraction, and Quantification

Buccal swab samples were collected from 144 Turkish male volunteers aged 50 years and older at the Andrology Outpatient Clinic, Department of Urology, Cerrahpaşa Faculty of Medicine, Istanbul University-Cerrahpaşa. The study was conducted in accordance with the Declaration of Helsinki, and written informed consent was obtained from all participants before enrolment. All procedures involving human participants were approved by the Clinical Research Ethics Committee of Istanbul University-Cerrahpaşa, Cerrahpaşa Faculty of Medicine (approval number 159145; 3 December 2020). Phenotype assessment was performed using the Hamilton–Norwood classification. Standardized scalp photographs were obtained under uniform lighting and at a consistent distance, with the vertex region visible, to support phenotype classification.

The analysis included 30 participants with HN grade 1 (no baldness) and 114 participants with HN grades 5–7 (severe MPB); intermediate HN grades were not included. For the training–test split, 86 participants were assigned to the training group (18 HN grade 1 and 68 HN grades 5–7) and 58 participants to the held-out test group (12 HN grade 1 and 46 HN grades 5–7). The prediction target was therefore restricted to discrimination between the two extreme phenotype categories rather than prediction across the full HN scale.

DNA was extracted using the E.Z.N.A.^®^ Tissue DNA Kit (Omega Bio-tek, Norcross, GA, USA) and quantified using the Qubit^®^ dsDNA High Sensitivity Assay (Thermo Fisher Scientific, Waltham, MA, USA) in accordance with the manufacturers’ instructions.

### 2.2. SNP Marker Selection and Primer Design

In this study, 23 candidate SNP markers were identified and selected following a comprehensive literature review and based on predefined criteria (Table 1). These criteria included consistent replication in large-scale GWAS and meta-analyses of male pattern baldness, proximity to genes involved in hair follicle biology, androgen signaling, and WNT/apoptosis pathways, appropriate minor allele frequencies (MAF > 1%) within the population, as evaluated using allele frequencies from the HapMap project and the 1000 Genomes Project Phase 3 database, low linkage disequilibrium (LD) to maximize independent genetic information, and technical feasibility for forensic casework. The selected markers are located both on the X chromosome, particularly within the *AR*/*EDA2R* locus on Xq12, and across multiple autosomal regions, including *PAX1*/*FOXA2*, *HDAC9*, *EBF1*, *TARDBP*, *AUTS2*, *SETBP1*, *HDAC4*, *WNT10A*, *SUCNR1*-*MBNL1*, *IRF4*, and *SSPN*-*ITPR2* (chromosomes 1, 2, 3, 5, 6, 7, 12, 18, and 20). Priority was given to loci, such as *AR*/*EDA2R*, 20p11/*PAX1*–*FOXA2*, and *WNT10A*, which previous predictive studies demonstrated to provide the highest incremental predictive power for severe MPB. 

Fasta sequences for the selected 23 SNP markers were obtained from the NCBI website (https://www.ncbi.nlm.nih.gov/) (accessed on 22 September 2026). PCR primers and SNaPshot primers for a multiplex study were designed using BatchPrimer3 v1.0 (https://probes.pw.usda.gov/cgi-bin/batchprimer3/batchprimer3.cgi) (accessed on 22 September 2026). The primers ranged in length from 17 to 25 base pairs, and the final panel amplicon lengths ranged from 69 to 153 base pairs. Primer melting temperatures were adjusted to 56–60 °C, maintaining a purine:pyrimidine ratio of 1:1. For SNaPshot primer design, the melting temperatures were set between 50–55 °C, and poly-T tails were added to differentiate among SNP markers based on size (Table 1).

Primers were evaluated using the AutoDimer web application (https://strbase.nist.gov/AutoDimerHomepage/DownloadPage.htm) (accessed on 22 September 2026) and BLAST (https://www.ncbi.nlm.nih.gov/tools/primer-blast/) (accessed on 22 September 2026) to screen for primer–dimer and hairpin interactions and to ensure primer specificity by identifying matching sequences within the genome was used—and SNPcheck tool (https://genetools.org/SNPCheck/snpcheck.htm) (accessed on 22 September 2026) was employed to verify that the designed primers did not contain any SNPs within the specific target sites. Table 1 illustrates details of the 23 SNPs, including SNaPshot primer amplicon sizes and observed polymorphisms. PCR and SNaPshot primer sequences are given in Supplementary Tables S1 and S2.

### 2.3. PCR, SNaPshot Reaction, and Capillary Electrophoresis

Multiplex PCR was performed in a total reaction volume of 10.0 μL containing 5.0 μL Master Mix (Applied Biosystems, Foster City, CA, USA), 1.0 μL nuclease-free water, 2.0 μL primer mix, and 2.0 μL DNA (approximately 2 ng). Amplification was performed using a SimpliAmp™ Thermal Cycler (Applied Biosystems, Foster City, CA, USA) with the following cycling conditions: an initial denaturation at 95 °C for 15 min, followed by 35 cycles of 94 °C for 30 s (denaturation), 58 °C for 90 s (annealing), and 72 °C for 90 s (extension), concluding with a final extension at 72 °C for 10 min. Amplicon presence was verified by agarose gel electrophoresis.

Following amplification, PCR products were purified using a standard purification protocol and incubated at 37 °C for 45 min, then at 80 °C for 15 min. For the SNaPshot multiplex reaction, 2.0 μL of purified PCR product was mixed with 1 μL SNaPshot Multiplex Kit (Thermo Fisher Scientific, Waltham, MA, USA), 1.0 μL nuclease-free water, and 1.0 μL SNaPshot primer mix, totalling a 5 μL reaction volume. This reaction underwent a cycle of 96 °C for 10 s (denaturation), 53 °C for 5 s (annealing), and 60 °C for 30 s (extension), for 30 cycles.

Final purification involved 1 μL rAPid alkaline phosphatase (Roche, Basel, Switzerland) at 37 °C for 80 min, followed by inactivation at 85 °C for 15 min. For capillary electrophoresis, 1.5 μL of the purified SNaPshot product was combined with 10 μL Hi-Di Formamide and 0.3 μL GeneScan-120 LIZ internal size standard (Thermo Fisher Scientific, Waltham, MA, USA). Separation and detection of SNaPshot products were performed using an ABI Prism^®^ 310 Genetic Analyzer (Thermo Fisher Scientific, Waltham, MA, USA) with POP4™ polymer. Allele detection was performed using GeneMapper v4.0 (Thermo Fisher Scientific, Waltham, MA, USA). Polymorphisms were assessed by electropherograms obtained for each SNP marker, and these genotypes were utilized to construct the Severe MPB prediction model.

### 2.4. Panel Optimization and Validation

Panel optimization was initiated with singleplex reactions using 2800 M control DNA (10 ng/µL) to determine the observed electrophoretic positions of the extension products. A multiplex primer mixture was subsequently prepared, and marker-specific primer concentrations were adjusted according to peak height and overall signal balance until all loci were consistently detected within an acceptable signal range.

Panel validation included assessment of the analytical threshold, dynamic range, sensitivity, and repeatability. The analytical threshold was established using 10 negative controls containing sterile distilled water. For dynamic range and sensitivity assessment, serial dilutions of 2800 M control DNA (10 ng/µL) were tested at DNA inputs of 2, 1, 0.5, 0.25, 0.125, and 0.05 ng per PCR reaction (Supplementary Figure S1). Repeatability was evaluated by reanalysis of five previously genotyped samples and 2800 M control DNA by an independent analyst under unchanged experimental conditions.

### 2.5. Statistical Analysis

#### 2.5.1. Prediction Modelling

Binary logistic regression analysis (BLRA) was used to develop the severe MPB prediction model. Genotype data from 86 individuals in the training group, comprising 18 participants with HN grade 1 and 68 participants with HN grades 5–7, were analyzed using univariable and multivariable BLRA in SPSS Statistics version 29.0 (IBM Corp., Armonk, NY, USA). Baldness status was entered as the dependent variable and coded as 0 for HN grade 1 (non-bald) and 1 for HN grades 5–7 (severe MPB). A total of 23 SNP markers were initially evaluated as independent variables. Autosomal SNPs were coded according to the number of target minor alleles (0, 1, or 2), whereas X-chromosomal SNPs were coded as 0 for the absence and 1 for the presence of the target minor allele. Odds ratios (ORs), corresponding 95% confidence intervals (CIs), regression coefficients, and *p*-values were estimated for the target minor alleles. Because the training set contained only 18 non-bald participants relative to the number of candidate predictors, the modelling was considered exploratory and the possibility of sparse-data bias, coefficient instability, and overfitting was explicitly considered when interpreting the results. The available cohort was therefore treated as suitable for proof-of-concept model development rather than for definitive forensic validation.

#### 2.5.2. Model Development and Predictor Selection

Model development and predictor selection were performed exclusively in the training set (*n* = 86; 18 participants with HN grade 1 and 68 with HN grades 5–7). The held-out test group (*n* = 58) was not used for univariable screening, assessment of marker correlations, predictor selection or removal, model specification, or coefficient estimation.

Three binary logistic regression analysis (BLRA) strategies were evaluated. Model 1 was fitted by simultaneously entering all 23 candidate SNP markers into a multivariable BLRA model (Supplementary Table S3). Although the full model showed high apparent discrimination in the training set, several regression coefficients were numerically unstable, with Wald statistics approaching zero and *p*-values approaching 1, suggesting overparameterization and possible separation. Model 2 was developed using forward conditional BLRA and retained two markers, rs7349332 (S02) and rs2497911 (S23) (Supplementary Table S7).

For the development of Model 3, each of the 23 candidate SNPs was initially evaluated using univariable BLRA in the training set. SNPs were ranked in ascending order of their univariable *p*-values and sequentially added to multivariable BLRA models to explore their incremental contribution to discrimination. After each successive marker addition, the apparent training-set area under the receiver operating characteristic curve (AUC) was calculated. This analysis was used descriptively to assess how the progressive inclusion of candidate markers affected model discrimination and was not used as the sole criterion for determining the final model.

Fourteen SNPs met the prespecified screening criterion of *p* < 0.25 in the univariable analyses and were retained for further multivariable evaluation (Supplementary Table S4). These markers were S01, S02, S03, S07, S10, S12, S17, S18, S19, S20, S21, S22, S24, and S25. The 14 markers were subsequently entered simultaneously into a multivariable BLRA model. Because numerical instability persisted, the correlation structure among the candidate predictors was examined.

Bivariate correlation analysis demonstrated strong intercorrelations among five X-chromosomal markers, S17, S20, S21, S22, and S24 (Supplementary Table S5). To reduce multicollinearity and improve coefficient stability, the five correlated markers were removed sequentially according to the documented historical model-development sequence, beginning with S22 and followed by S21, S17, S20, and S24. The multivariable BLRA model was re-estimated after each removal. This sequential correlation-guided reduction yielded the final nine-marker Model 3 comprising S01 (rs12565727), S02 (rs7349332), S03 (rs9287638), S07 (rs2073963), S10 (rs9668810), S12 (rs10502861), S18 (rs1041668), S19 (rs1511061), and S25 (rs16990427).

Model 3 was retained as the principal exploratory prediction model because it reduced marker redundancy and the numerical instability observed in the larger multivariable specifications while maintaining a more parsimonious set of predictors from multiple MPB-associated loci. Apparent training-set AUC was considered together with coefficient stability, multicollinearity, and model complexity rather than being used as an isolated model-selection criterion. Once the final nine-marker specification had been established, no additional predictors were removed solely on the basis of individual Wald *p*-values. Accordingly, the retained coefficients were interpreted primarily as components of a multivariable prediction model rather than as evidence that each SNP represented an independently significant association. 

To further contextualize the retention of the final marker set, an additional descriptive analysis was performed after the nine-SNP specification had been fixed. In the training set only, rs7349332 (S02)—the sole predictor retaining statistical significance in the final multivariable model—was evaluated as a single-predictor classifier, and its apparent AUC was compared with that of the fixed nine-SNP Model 3. This comparison was not used to select or remove predictors, and no held-out test-group data were used for this purpose.

#### 2.5.3. Model Validation

All three models obtained by BLRA were evaluated in a held-out test group of 58 volunteers derived from the same overall cohort, comprising 12 participants with HN grade 1 and 46 with HN grades 5–7. Accordingly, this analysis represents internal split-sample validation and not true external validation. Prediction probabilities and AUC values were calculated for each model. Diagnostic performance metrics in the held-out test group were calculated at both the conventional threshold of 0.50 and the model-specific optimized threshold. The optimized thresholds were determined in the training group and then evaluated in the held-out test group. In addition, fivefold cross-validation was performed using the complete cohort (*n* = 144; 30 HN grade 1 and 114 HN grades 5–7). The stratified fivefold partition was generated using a fixed random seed of 42 and was kept identical across the three model specifications. Within each fold, regression coefficients for the predefined model specification were re-estimated using only the corresponding training folds and then applied to the validation fold. SNP selection was not repeated within each fold. Prediction probabilities from the five validation iterations were combined to calculate an overall out-of-fold AUC, with a bootstrap 95% CI. Cross-validation was therefore interpreted as internal validation of the fixed model specifications rather than validation of the entire historical marker-selection process.

#### 2.5.4. Calibration Assessment

Calibration of the final 9-SNP model was evaluated in the 58-person held-out test group. Predicted probabilities from the model fitted in the 86-person training group were applied without refitting. Overall prediction error was summarized using the Brier score. Calibration intercept and slope were estimated by logistic recalibration of the observed outcome on the logit of the predicted probability; an intercept of 0 and a slope of 1 indicate ideal calibration. Calibration-in-the-large was also calculated as the difference on the logit scale between the observed event frequency and the mean predicted probability. A calibration plot was constructed using grouped observed proportions together with the logistic calibration curve. Because the held-out test group was small, calibration estimates were interpreted descriptively and with particular attention to overfitting.

## 3. Results

### 3.1. Optimisation of the 23-SNP Panel

Each SNP in the 23-SNP panel was initially analyzed as a singleplex by capillary electrophoresis using 2800 M control DNA. Differences between expected and observed product lengths were recorded because dye binding, primer length, and nucleotide composition can affect electrophoretic mobility. The expected and observed SNaPshot product sizes for the 23 SNPs are provided in Supplementary Table S6. To optimize the 23-SNP multiplex panel, primer concentrations were adjusted between 0.125 μM and 4.0 μM to balance high and low peak intensities. Figure 1 shows the optimized electropherogram of the 23-SNP panel.

### 3.2. Validation of the 23-SNP Panel

The analysis threshold was set at 80.37 RFU based on the results from ten negative controls. When evaluating electropherogram images of 2800 M control DNA at various amounts (0.05, 0.125, 0.25, 0.5, 1, and 2 ng), a complete DNA profile was achieved with DNA inputs ranging from 0.25 to 2.0 ng. Allelic dropouts were observed at lower DNA amounts, specifically at 0.125 ng for the S13 and at 0.05 ng for the S14 (Supplementary Figure S1). In the sensitivity analysis, complete profiles were consistently observed at the lowest DNA amounts of 0.25 ng and 0.5 ng. The Limit of Quantitation (LOQ) was established at 0.5 ng by calculating the mean and standard deviation of peak heights for each allele at these DNA amounts.

A complete DNA profile was achieved with a minimum DNA amount of 0.25 ng, while a complete and reliable profile required a minimum input of 0.5 ng. Given the often limited and degraded condition of biological evidence in forensic cases, these DNA inputs were considered suitable for the study. Furthermore, the consistent detection of identical genotypes in the electropherograms of 2800 M control DNA, analyzed using the 23-SNP panel by different analysts under unchanged experimental conditions, confirmed the success of the reproducibility study.

### 3.3. Statistical Modelling and Validation

In the univariable BLRA, 14 of the 23 candidate SNP markers met the prespecified screening criterion of *p* < 0.25 and were retained for exploratory multivariable modelling (Supplementary Table S4). When the SNPs were sequentially added to multivariable BLRA models according to their univariable statistical evidence, apparent discrimination in the training set showed an overall increase, although the improvement was not monotonic at every step. The model containing the 14 screened SNPs yielded an apparent training-set AUC of 0.899. Inclusion of the remaining candidate markers increased the apparent AUC to 0.941 for the full 23-SNP Model 1. However, the full model showed substantial numerical instability, including Wald statistics approaching zero, *p*-values approaching 1, and unstable coefficient estimates. Thus, the higher apparent training-set AUC of the full model was not considered sufficient evidence for selecting it as the final prediction model.

Persistent numerical instability in the 14-marker multivariable model prompted evaluation of the correlation structure among the candidate predictors. Five X-chromosomal markers, S17, S20, S21, S22, and S24, showed strong intercorrelations (Supplementary Table S5). To reduce multicollinearity, these markers were removed sequentially according to the historical model-development sequence documented in the original analysis: S22 was removed first, followed by S21, S17, S20, and S24. After each removal, the multivariable BLRA model was re-estimated using the remaining predictors. This correlation-guided reduction yielded the final nine-marker Model 3 comprising S01, S02, S03, S07, S10, S12, S18, S19, and S25. Model 3 was retained as the principal exploratory model because it reduced marker redundancy and coefficient instability while providing a more parsimonious multivariable specification. Accordingly, the final model was retained after considering apparent discrimination, coefficient stability, multicollinearity, and model parsimony; no single performance measure was used as the sole model-selection criterion. The held-out test group was not used for SNP screening, marker removal, model specification, or model choice. 

The performance characteristics of the three prespecified modelling approaches are summarized in Table 2. Model 1 achieved the highest apparent training-set AUC (0.941) but showed substantial coefficient instability. Model 2, derived by forward conditional BLRA, retained rs7349332 (S02) and rs2497911 (S23) and yielded a training-set AUC of 0.834. The final nine-marker Model 3 yielded a training-set AUC of 0.879. For Model 3, the Hosmer–Lemeshow test was χ^2^ = 5.922 (*p* = 0.549), and the Nagelkerke R^2^ was 0.466. These apparent training-set measures were interpreted together with the subsequent held-out and cross-validation results rather than as evidence of external validation.

Receiver operating characteristic curves for Models 1–3 in the training and held-out test groups are presented in Figure 2. The training AUCs were 0.941, 0.834, and 0.879 for Models 1, 2, and 3, respectively; the corresponding held-out test AUCs were 0.895, 0.739, and 0.777 (Table 2). For Model 3, the optimized cut-off determined by the Youden and Liu indices was 0.843. The decrease from training to held-out test performance, particularly for the more complex Model 1, was interpreted as evidence that apparent training performance may overestimate generalizable discrimination.

For Model 3, the overall correct classification rates in the training group were 87.2% and 75.6% at thresholds of 0.50 and 0.843, respectively. At 0.50, high sensitivity was observed (0.985), whereas the optimized threshold of 0.843 increased specificity to 0.944. In the held-out test group, sensitivity and specificity were 0.935 and 0.250 at 0.50 and 0.717 and 0.750 at 0.843, respectively (Table 2). These operating characteristics are conditional on the study’s deliberately enriched extreme-phenotype sample and should not be interpreted as population-level estimates. In particular, PPV and NPV are prevalence-dependent, and the optimized threshold of 0.843 is a cohort-derived operating point rather than a validated forensic decision threshold.

Additional fivefold cross-validation was performed for all three prediction models. The overall cross-validated AUCs were 0.757 (bootstrap 95% CI, 0.642–0.861) for Model 1, 0.766 (bootstrap 95% CI, 0.664–0.861) for Model 2, and 0.786 (bootstrap 95% CI, 0.696–0.868) for Model 3. The corresponding mean fold AUCs were 0.761 ± 0.049, 0.795 ± 0.070, and 0.786 ± 0.098, respectively. For each predefined model specification, regression coefficients were re-estimated within the training folds and out-of-fold probabilities were generated for the omitted fold; marker screening and correlation-guided selection were not repeated within folds. Fold-level and overall cross-validation results are summarized in Table 3, and pooled out-of-fold ROC curves for all three model specifications are shown in Figure 3. Thus, the cross-validation analysis estimates the internal performance of the fixed model specifications and does not constitute selection-adjusted or external validation.

Calibration of Model 3 was additionally evaluated in the 58-person held-out test group. The Brier score was 0.159. The calibration intercept was 0.568 and the calibration slope was 0.492; calibration-in-the-large was −0.033 because the mean predicted probability (0.797) was close to the observed severe-MPB proportion (0.793). The slope below 1 indicates that the predicted probabilities were too extreme, consistent with residual overfitting. Because only 12 non-bald participants were present in the held-out test group, the calibration estimates were imprecise and should be interpreted as descriptive internal-validation results rather than evidence of transportability (Figure 4).

The probability equation for severe MPB was based on the regression coefficients of the nine predictors and the constant term shown in Table 4. The coefficients represent model weights and should be interpreted as components of the multivariable prediction equation rather than as evidence that every retained SNP has an independently significant biological effect.

P (severe MPB) = 1/[1 + exp(−z)]

z = 1.200 + 0.703(S01) + 1.743(S02) + 0.689(S03) + 0.835(S07) − 0.876(S10) − 0.905(S12) − 1.881(S18) − 1.807(S19) + 0.738(S25)

For descriptive classification, probabilities greater than 0.50 were classified as severe MPB and probabilities below 0.50 as non-bald. The threshold of 0.843 was evaluated as an additional cohort-derived operating point selected from the training ROC analysis. Neither threshold should be regarded as a population-independent forensic cut-off without external validation, particularly because intermediate HN grades were excluded from model development.

Among the nine predictors included in Model 3, rs7349332 (S02), located in the *WNT10A* region, was the only marker that remained statistically significant after simultaneous adjustment (Table 4). Each additional copy of the target A allele was associated with approximately 5.7-fold higher odds of severe baldness. The remaining eight markers were retained as components of the fixed multivariable prediction equation because they arose from the training-only marker-reduction process and represented joint predictive information across seven loci; however, they did not demonstrate statistically significant independent associations after adjustment. Their coefficients should therefore not be interpreted as isolated causal effects. As a descriptive training-set comparison performed after the nine-marker specification had been fixed, rs7349332 alone yielded an AUC of 0.691, compared with 0.879 for the complete nine-SNP Model 3 (absolute difference, 0.188). This supports additional apparent discrimination from the combined marker set beyond rs7349332 alone, despite the other eight coefficients not reaching individual statistical significance after adjustment. Because this comparison was performed in the development data, it was treated as supportive rather than independent validation evidence and was not used to redefine the model.

## 4. Discussion

In this study, a 23-SNP panel across twelve MPB-associated loci—*AR*/*EDA2R*, *PAX1*/*FOXA2*, *HDAC4*, *HDAC9*, *TARDBP*, *AUTS2*, *SETBP1*, *WNT10A*, *SUCNR1*-*MBNL1*, *EBF1*, *SSPN*-*ITPR2*, and *IRF4*—was optimized and evaluated in 144 Turkish males aged 50 years and older. Participants were classified into two extreme phenotype groups: HN grade 1 (no baldness) and HN grades 5–7 (severe baldness). Genotyping was performed using the SNaPshot method, and a final 9-SNP severe-MPB prediction model was developed using binary logistic regression. The final model comprised rs12565727 (*TARDBP*), rs7349332 (*WNT10A*), rs9287638 (*HDAC4*), rs2073963 (*HDAC9*), rs9668810 (*SSPN*–*ITPR2* region), rs10502861 (*SETBP1*), and three X-chromosomal SNPs—rs1041668, rs1511061, and rs16990427—within the *AR*/*EDA2R* region. The model yielded an AUC of 0.879 in the training group and 0.777 in the held-out test group derived from the same cohort. These findings represent internal evaluation of an exploratory candidate model rather than external validation.

### 4.1. Statistical Analyses and Model Development

The present study aimed to evaluate associations between selected SNP genotypes and severe MPB and to develop an SNP-based prediction model using multivariable logistic regression. The final 9-marker model showed improved Wald statistics and more stable confidence intervals than the preliminary multivariable models. However, bootstrap evaluation indicated substantial sampling uncertainty, and marker-level effects were therefore interpreted cautiously in relation to the previous literature and biological plausibility.

In univariable logistic regression, 14 SNPs met the prespecified screening criterion of *p* < 0.25, and 10 showed statistically significant associations with severe MPB at *p* < 0.05. Eight of these signals were located in the highly correlated *AR*/*EDA2R* region on Xq12, whereas the remaining two were rs12565727 at 1p36.22 (*TARDBP*) and rs7349332 at 2q35 (*WNT10A*). In the multivariable model, most associations were attenuated and rs7349332 remained the only statistically significant independent predictor. Previous MPB prediction studies have also reported predictive relevance for rs7349332 [39,52,57]. *WNT10A* has a functional role in hair-follicle cycling, and reduced expression has been proposed to delay the telogen-to-anagen transition and shorten anagen [35,36]. Three additional retained markers—rs1041668, rs1511061, and rs16990427—were located within the *AR*/*EDA2R* region on the X chromosome. Previous studies consistently support a major contribution of Xq12 to MPB susceptibility [39,49,52,53,58,59], while experimental work has reported higher *EDA2R* expression in balding dermal papilla cells than in non-balding cells [56]. Subsequent studies have continued to support the importance of the *AR*/*EDA2R* region in MPB [57]. Consistent with the predictive rather than purely inferential role of the retained marker set, rs7349332 alone provided an apparent training-set AUC of 0.691, whereas the fixed nine-SNP model yielded 0.879. Thus, lack of individual statistical significance for the other retained coefficients should not be equated with absence of predictive information at the multivariable model level. Nevertheless, this development-set comparison may be optimistic and requires confirmation in an external cohort. For classification, the model-specific operating threshold was derived from training-set ROC analysis using the Youden and Liu criteria, established approaches to cut-point selection [60,61].

A notable feature of the Xq12 markers was the direction of effect observed for the target minor alleles, which generally corresponded to lower odds of severe MPB in the univariable analyses. Similar inverse associations for several minor alleles have been reported previously [39,53,62]. Because these X-chromosomal markers are correlated and the training sample contained only 18 non-bald participants, adjusted marker-specific coefficients are particularly susceptible to sampling variation and should not be interpreted as stable causal effect estimates. Broader genetic reviews and association studies have likewise emphasized heterogeneity across *AR*/*EDA2R* and additional susceptibility loci [63,64].

Beyond Xq12, the *TARDBP* locus (1p36.22) and *WNT10A* locus (2q35) were represented in the final model by rs12565727 and rs7349332, respectively. *TARDBP* encodes TAR DNA-binding protein 43 (TDP-43), which has multiple roles in transcription, mRNA processing, and microRNA regulation. Although its relationship to alopecia remains unclear, *TARDBP* variants have been implicated in amyotrophic lateral sclerosis (ALS) [65], early-onset alopecia has been associated with ALS risk [66], and additional *TARDBP* sequence analyses have identified disease-associated mutations [67]. By contrast, *WNT10A* has been repeatedly associated with MPB and has a biologically plausible role in initiation and maintenance of the anagen phase [35,39,52,57].

Histone deacetylase proteins (HDAC4 at 2q37 and HDAC9 at 7p21.1) also play an important role in *AR* signalling and hair follicle miniaturization, key features of AGA [46,51]. SNPs from the *HDAC4* locus (rs9287638) and *HDAC9* locus (rs2073963, rs756853) were evaluated, with all except rs756853 included in our main model. 

Two additional SNPs retained in the 9-SNP model were rs9668810, located in the *SSPN*–*ITPR2* region, and rs10502861, located near *SETBP1*. Previous work has reported expression of genes in these regions in hair-related tissues, although the functional mechanisms linking these loci to MPB remain incompletely defined [51]. Li et al. identified rs10502861 among MPB-associated loci and provided replication evidence across European population samples [51].

### 4.2. Comparison with Previous Prediction Models, Strengths, and Limitations

Several prediction models for male pattern baldness have previously been developed. Marcińska et al. developed 5-SNP and 20-SNP models in a cohort of 605 European men and reported AUC values of 0.762 and 0.864, respectively, for distinguishing men younger than 50 years with severe baldness from older men without baldness [39]. Five SNPs included in their 20-SNP model, rs12565727, rs7349332, rs9668810, rs10502861, and rs1041668, were also retained in our 9-SNP model. In the present study, AUC values of 0.879 in the training group and 0.777 in the held-out test group were obtained, despite the use of a smaller marker set and a study population restricted to men aged 50 years and older. The overlap in the five selected markers provides additional support for the potential predictive relevance of these loci in the Turkish cohort. However, direct comparison between the models should be made cautiously because of differences in age distribution, phenotype definition, population background, marker composition, and statistical design. The use of a reduced 9-SNP panel may nevertheless offer a more parsimonious approach to MPB prediction and could be advantageous for forensic DNA phenotyping, particularly when limited amounts of DNA are available. Further validation in larger and independent populations is required to determine the generalizability and forensic utility of the model.

Similarly, Liu et al. used various SNP sets (6-SNPs, 11-SNPs and 14-SNPs) across three different cohorts in 2725 German and Dutch men [52]. An AUC of 0.711, with a sensitivity of 0.73 and a specificity of 0.58, was reported for an 11-SNP-plus-age model used to distinguish no baldness from any degree of baldness in older men. Differences in phenotype definition are particularly important, because the present study was restricted to HN grade 1 versus HN grades 5–7 and therefore represents a more distinct phenotype contrast. 

Later studies employed substantially larger cohorts and more comprehensive genetic marker panels. Hagenaars et al. developed a prediction model based on 287 SNPs using data from more than 40,000 UK Biobank participants aged 40–69 years [53]. The model was subsequently validated in an additional 12,874 UK Biobank participants and achieved an AUC of 0.78 for distinguishing no baldness from severe baldness. Chen et al. developed MPB prediction models based on 117 SNPs using binary and multinomial logistic regression as well as machine-learning approaches [57]. The models were evaluated in 26,177 independent samples. Without age as a predictor, AUC values of 0.718, 0.607, 0.574, and 0.702 were reported for severe, moderate, slight, and no baldness, respectively. When age was included, the corresponding AUC values increased to 0.728, 0.635, 0.602, and 0.711. In their binary logistic-regression model for distinguishing any degree of baldness from no baldness, AUCs of 0.702 without age and 0.711 with age were obtained. These findings indicate that age provided only a modest improvement in discrimination and also highlight the difficulty of predicting intermediate baldness phenotypes compared with more distinct phenotype categories. In contrast, the present study focused on the more clearly separated phenotype categories of HN grade 1 and HN grades 5–7, which should be considered when comparing predictive performance across studies.

Several practical strengths were present in the study design. All participants were men aged 50 years or older, the prediction target was explicitly defined, standardized phenotype documentation was used, and SNP genotyping was performed using a multiplex assay suitable for relatively low DNA inputs. In addition, the prediction equation was evaluated in the held-out test group derived from the same cohort and further assessed by fivefold cross-validation using the complete cohort.

Despite these strengths, the marker-level findings from the multivariable analyses should be interpreted cautiously because of the limited sample size, marked class imbalance, and the large number of candidate predictors relative to the 18 non-bald participants in the training set. The unstable coefficients, zero Wald statistics observed in the full 23-SNP model, broad confidence intervals, and calibration slope below 1 in the held-out test group collectively indicate a material risk of overfitting. The attenuation of most single-marker associations after multivariable adjustment may reflect both sampling variability and the correlated, polygenic architecture of MPB. Accordingly, the present sample should be regarded as supporting exploratory proof-of-concept modelling rather than definitive estimation of marker effects or a fully validated forensic prediction system. Larger datasets and penalized or otherwise regularized modelling strategies may provide more stable estimates, but external validation remains essential. In larger datasets, multivariable approaches that explicitly accommodate correlated predictors, including network-regression frameworks, may also warrant evaluation [68].

Studies in Asian, European, and African populations indicate that the distribution and predictive effects of MPB-associated variants can differ across populations. Lower linkage disequilibrium in the *AR*/*EDA2R* region has been reported in African populations [69]. Kim et al. found that several *AR*/*EDA2R* variants identified in European and Chinese studies were not replicated in a Korean population, whereas the 20p11 locus showed more consistent evidence across populations [70]. In the present Turkish cohort, none of the 20p11 markers evaluated, including rs2180439, was retained in the final model, illustrating a possible population-specific difference that requires confirmation in larger samples. More recently, Janivara et al. evaluated 2136 African men and found that polygenic scores derived from European GWAS data yielded AUC values of only 0.513–0.546, providing direct evidence of limited cross-ancestry transferability for MPB prediction [71]. A recent genetic review similarly emphasizes both shared and population-specific MPB susceptibility signals and the need for ancestry-diverse validation [72].

An additional limitation is the extreme-phenotype design. The model was developed only in men aged 50 years or older and deliberately contrasted HN grade 1 with HN grades 5–7, excluding intermediate grades. This design can increase apparent discrimination by maximizing phenotypic separation and therefore may yield AUC, sensitivity, specificity, and optimized-threshold estimates that are more favorable than would be expected in an unselected forensic population. The reported PPV and NPV are also conditional on the enriched prevalence of severe MPB in this study and are not directly transportable to populations with different phenotype distributions. The model should therefore not be used to predict HN grades 2–4, and the 0.843 operating threshold should be considered study-specific. Future studies should include intermediate grades, broader age ranges, more balanced phenotype distributions, and independent external cohorts.

## 5. Conclusions

In this study, a 9-SNP model comprising markers from seven MPB-associated loci was developed to distinguish severe male pattern baldness (HN grades 5–7) from no baldness (HN grade 1) in Turkish males aged 50 years and older. The model showed moderate discrimination, with an AUC of 0.777 in the held-out test group derived from the same cohort and an overall fivefold cross-validated AUC of 0.786. Calibration analysis in the held-out test group yielded a Brier score of 0.159 and a calibration slope of 0.492, indicating that probability estimates remained overconfident. Among the evaluated markers, rs7349332 at the *WNT10A* locus was the most consistent individual predictor, whereas the remaining markers should be interpreted primarily as components of the multivariable prediction equation.

The proposed model should be considered a candidate, proof-of-concept forensic DNA phenotyping model rather than a fully validated forensic prediction system. Its small and imbalanced training sample, extreme-phenotype design, exclusion of intermediate HN grades, and validation only within the same overall cohort limit current generalizability. The reported classification metrics and cohort-derived thresholds should therefore be interpreted cautiously and should not be used as established forensic decision rules. External validation in larger independent Turkish and ancestry-diverse cohorts, with broader age ranges and full HN phenotype representation, is required before forensic implementation. Following such validation, genetic prediction of severe MPB may provide supplementary investigative information when conventional DNA profiling does not identify a suspect, while its probabilistic and population-dependent nature remains central to interpretation.

## Figures and Tables

**Figure 1:**
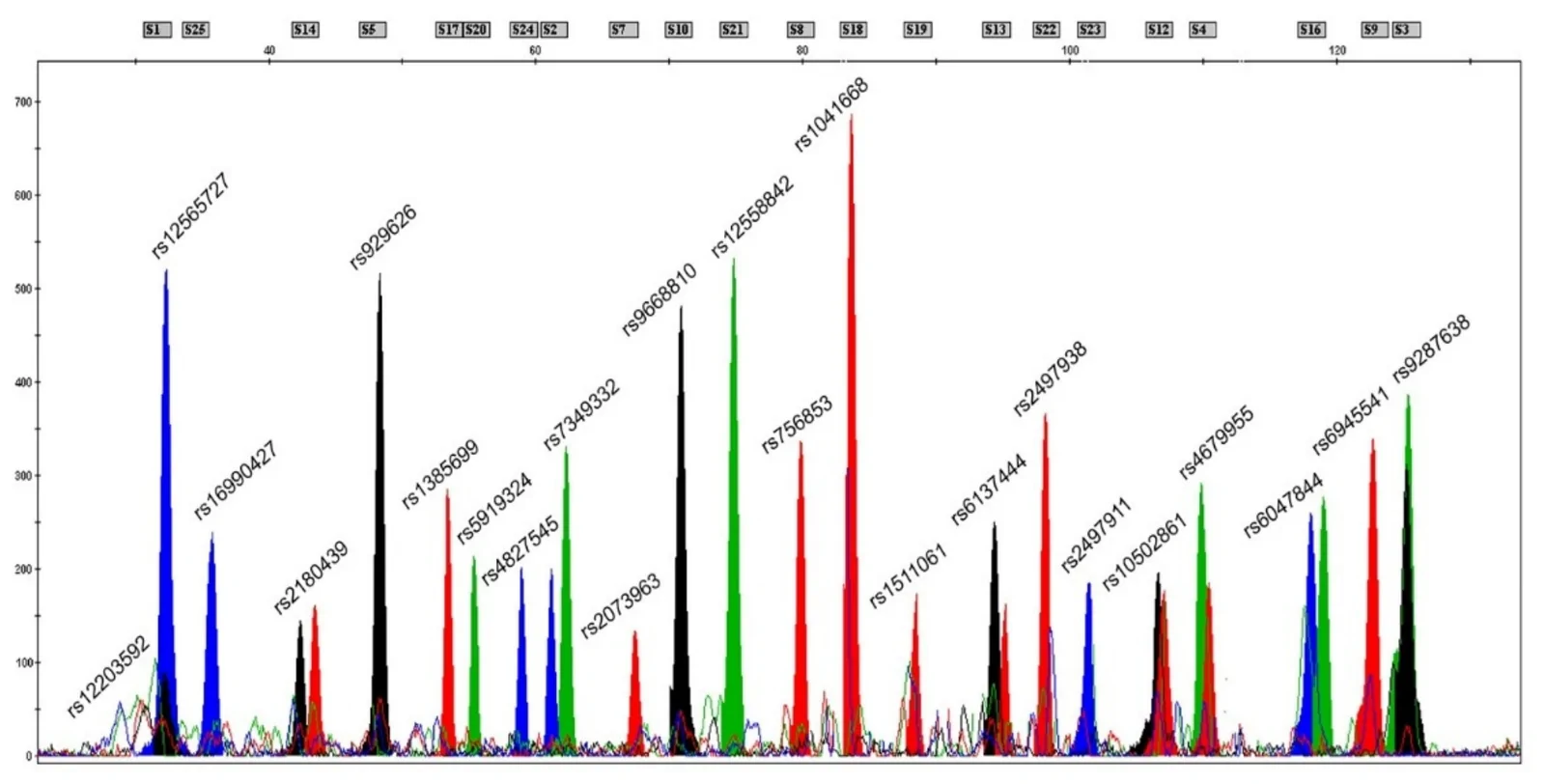
Optimized electropherogram of the 23-SNP SNaPshot multiplex panel generated with 2800 M control DNA. Colored peaks represent the fluorescent signals of the detected SNaPshot extension products.

**Figure 2:**
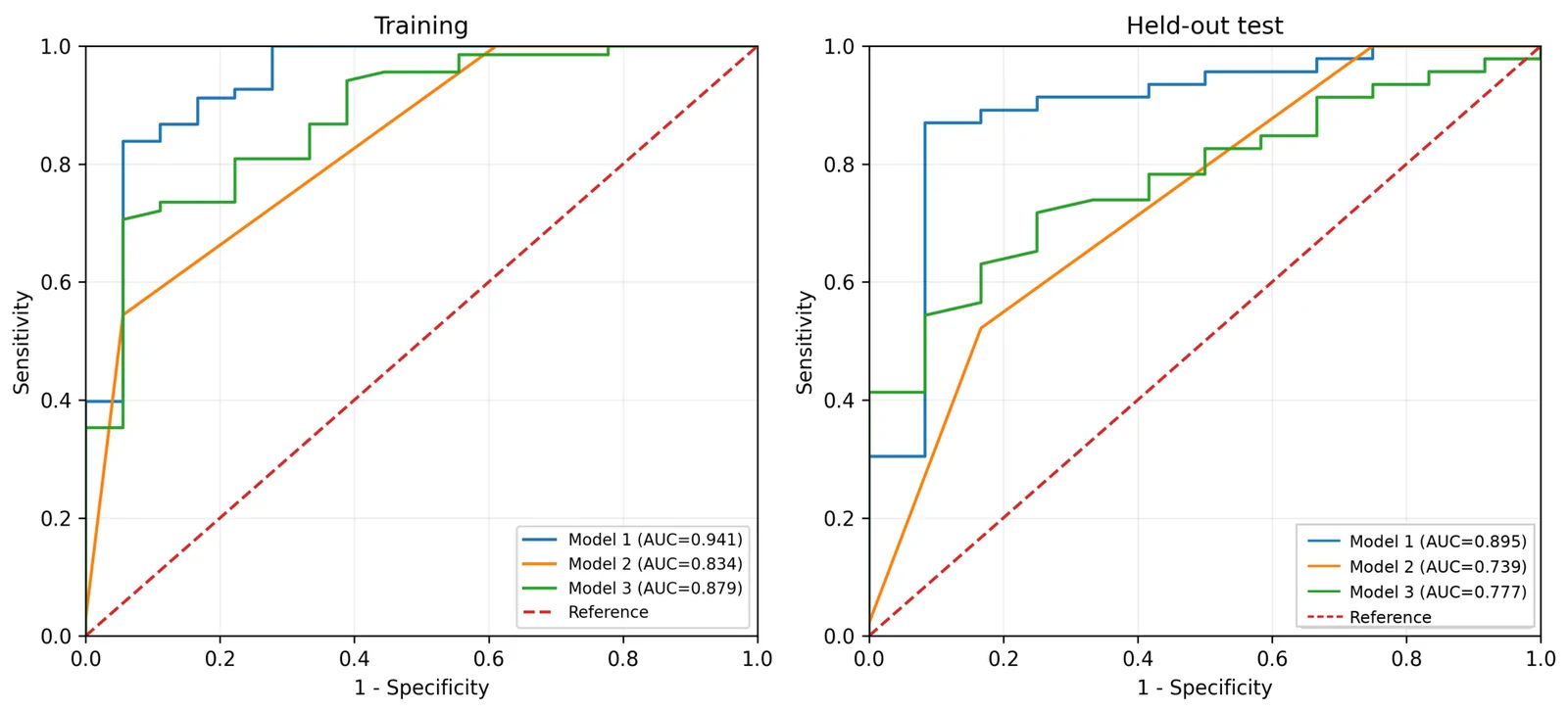
Receiver operating characteristic (ROC) curves for Models 1–3 in the training group (**left**) and the held-out test group derived from the same cohort (**right**). Training AUCs were 0.941, 0.834, and 0.879, and held-out test AUCs were 0.895, 0.739, and 0.777 for Models 1, 2, and 3, respectively. The dashed diagonal line indicates chance-level discrimination.

**Figure 3:**
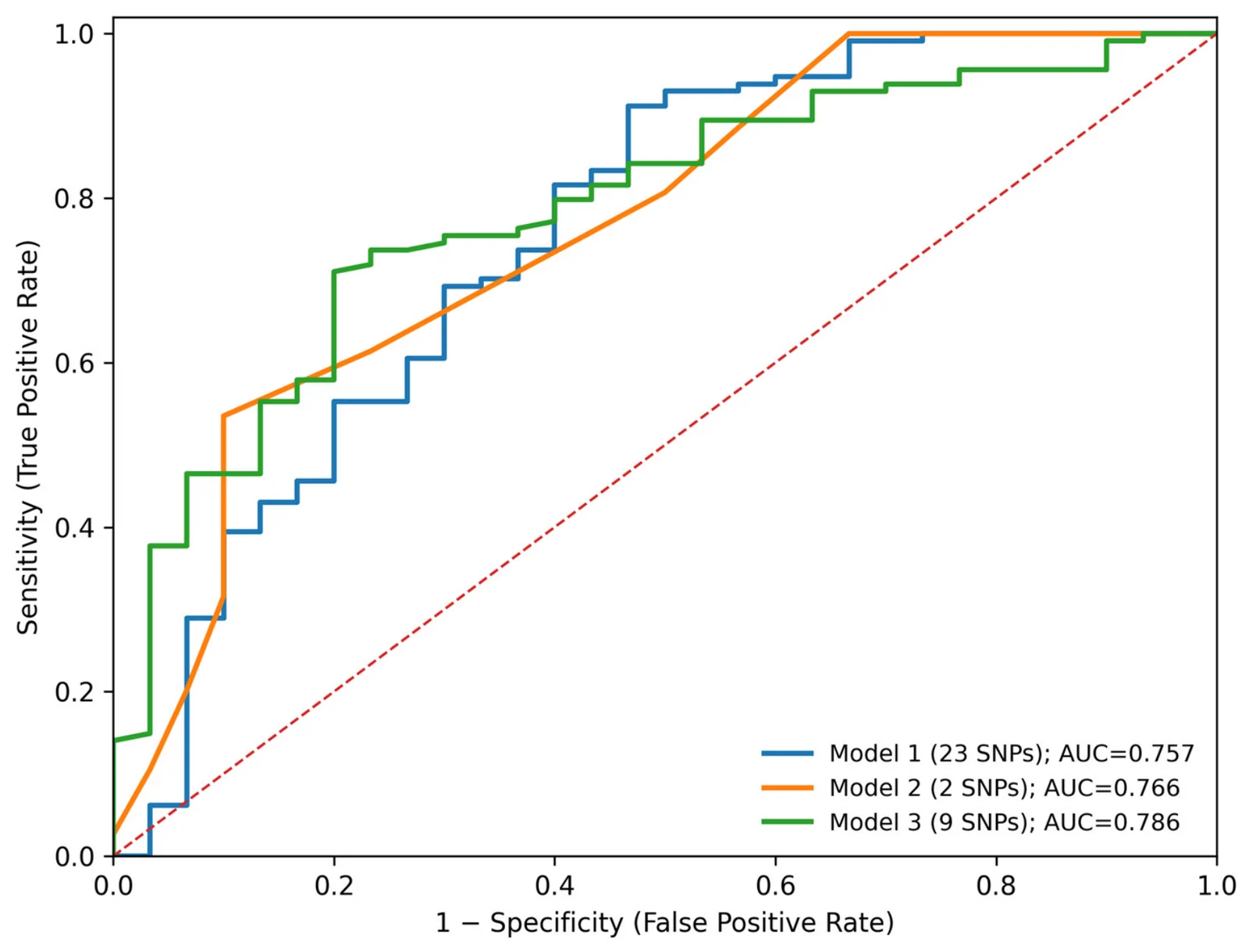
Pooled out-of-fold receiver operating characteristic (ROC) curves for the fixed Model 1 (23 SNPs), Model 2 (2 SNPs), and Model 3 (9 SNPs) specifications during stratified fivefold cross-validation of the complete cohort (*n* = 144). Overall cross-validated AUCs were 0.757, 0.766, and 0.786, respectively. Regression coefficients were re-estimated within each training fold; marker selection was not repeated within folds. The dotted diagonal line indicates chance-level discrimination.

**Figure 4:**
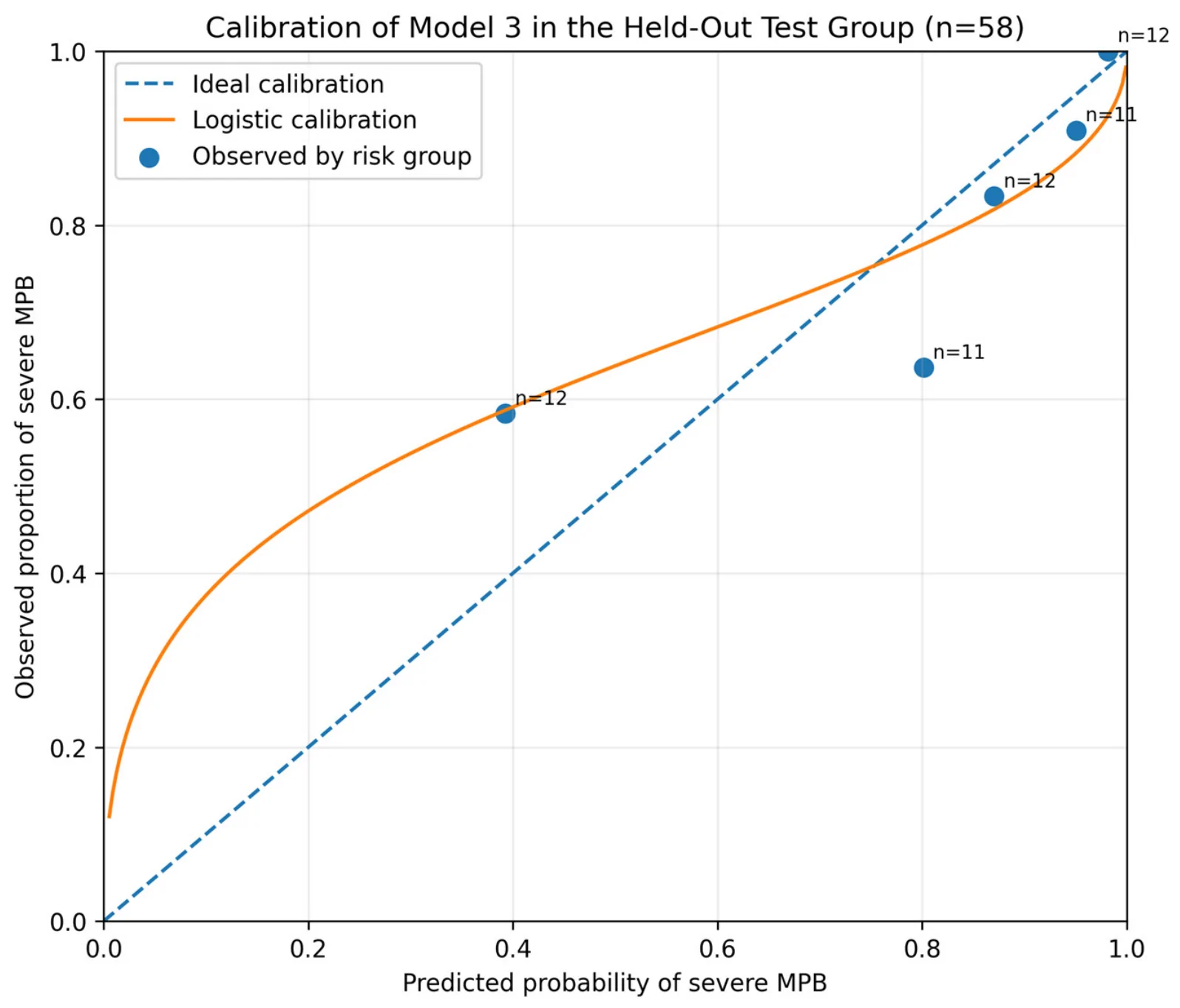
Calibration of the final 9-SNP Model 3 in the 58-person held-out test group. The Brier score was 0.159, calibration intercept 0.568, calibration slope 0.492, and calibration-in-the-large −0.033. The dashed line represents ideal calibration, the solid curve represents logistic recalibration, and points show observed severe-MPB proportions across grouped predicted risks.

**Table 1:** The 23 SNP markers included in the MPB multiplex panel, with gene regions, chromosomal positions, amplicon lengths, polymorphisms, minor alleles, and supporting references.

SNP ID	SNP Lab Code	Gene Region	Chromosomal Positions	Primer Amplicon Length (bp)	SNP	Minor Allele	References
rs12565727	S01	*TARDBP (C1orf127)*	chr1: 10973025	74	A/G	G	[39,51,52]
rs7349332	S02	*WNT10A*	chr2: 218891661	101	G/A	A	[35,39,43]
rs9287638	S03	*HDAC4*	chr2: 238785990	112	C/A	A	[51]
rs4679955	S04	*SUCNR1*–*MBNL1*	chr3: 151935580	112	A/T	T	[35,39]
rs929626	S05	*EBF1*	chr5: 158883623	88	T/C	C	[35,39]
rs12203592	S06	*IRF4*	chr6: 396321	120	C/T	T	[54]
rs2073963	S07	*HDAC9*	chr7: 18838251	81	T/G	G	[54]
rs756853	S08	*HDAC9*	chr7: 18850377	117	C/T	T	[39,46]
rs6945541	S09	*AUTS2*	chr7: 69146973	114	T/C	C	[39,51,52]
rs9668810	S10	*SSPN-ITPR2*	chr12: 26273487	108	T/C	C	[35,39]
rs10502861	S12	*SETBP1 (SLC14A2)*	chr18: 45220183	99	C/T	T	[39,51,52]
rs6137444	S13	*-*	chr20: 21805001	108	C/T	T	[31]
rs2180439	S14	*-*	chr20: 21872462	115	T/C	C	[31,39,46,53]
rs6047844	S16	*PAX1*/*FOXA2*	chr20: 22056937	69	G/A	A	[51]
rs1385699	S17	*EDA2R*	chrX: 66605144	115	T/C	C	[39,51,58,59]
rs1041668	S18	*AR*	chrX: 66866114	107	T/C	C	[39]
rs1511061	S19	*AR*	chrX: 67096282	80	T/C	C	[52]
rs5919324	S20	*AR/EDA2R*	chrX: 67210798	153	A/G	G	[39]
rs12558842	S21	*EDA2R*	chrX: 67261958	116	A/C	C	[53]
rs2497938	S22	*AR*	chrX: 67343176	111	T/C	C	[46,51,52]
rs2497911	S23	*AR/EDA2R*	chrX: 67407598	107	G/T	T	[39,51,54,58]
rs4827545	S24	*AR*	chrX: 67608543	115	G/A	A	[39,49,51,58]
rs16990427	S25	*-*	chrX: 67953948	80	G/A	A	[39,49,51,58]

**Table 2:** Discrimination and classification performance of Models 1–3 in the training and held-out test groups at the conventional threshold (0.50) and the model-specific training-derived optimized thresholds.

Model	Cohort	AUC	Threshold	Sensitivity	Specificity	PPV	NPV	Accuracy	F1
Model 1	Training	0.941	0.50	1.000	0.666	0.919	1.000	0.930	0.958
	Held-out test group	0.895	0.50	0.848	0.583	0.886	0.500	0.793	0.866
	Training	0.941	0.833	0.838	0.944	0.983	0.607	0.860	0.904
	Held-out test group	0.895	0.833	0.826	0.917	0.974	0.579	0.845	0.894
Model 2	Training	0.834	0.50	1.000	0.389	0.861	1.000	0.872	0.925
	Held-out test group	0.739	0.50	1.000	0.250	0.836	1.000	0.845	0.910
	Training	0.834	0.864	0.544	0.944	0.974	0.354	0.628	0.698
	Held-out test group	0.739	0.864	0.522	0.833	0.923	0.3125	0.586	0.666
Model 3	Training	0.879	0.50	0.985	0.444	0.870	0.889	0.872	0.924
	Held-out test group	0.777	0.50	0.935	0.250	0.827	0.500	0.793	0.877
	Training	0.879	0.843	0.706	0.944	0.980	0.459	0.756	0.820
	Held-out test group	0.777	0.843	0.717	0.750	0.917	0.409	0.724	0.804

**Model fit statistics:** Model 1, Nagelkerke R^2^ = 0.682; Hosmer–Lemeshow χ^2^ = 11.553, *p* = 0.116; AIC = 86.791. Model 2, Nagelkerke R^2^ = 0.503; Hosmer–Lemeshow χ^2^ = 0.000, *p* = 0.996; AIC = 60.703. Model 3, Nagelkerke R^2^ = 0.466; Hosmer–Lemeshow χ^2^ = 5.922, *p* = 0.549; AIC = 77.710. Abbreviations: AIC, Akaike information criterion; AUC, area under the receiver operating characteristic curve; NPV, negative predictive value; PPV, positive predictive value. Training rows are shaded. Model-specific optimized thresholds were 0.833, 0.864, and 0.843 for Models 1, 2, and 3, respectively. Each optimized threshold was determined from the training-group ROC analysis and then applied unchanged to the held-out test group.

**Table 3:** Fivefold cross-validation performance of the fixed Model 1 (23 SNPs), Model 2 (2 SNPs), and Model 3 (9 SNPs) specifications using the complete cohort (*n* = 144).

Model	No. of SNPs	Fold 1 AUC	Fold 2 AUC	Fold 3 AUC	Fold 4 AUC	Fold 5 AUC	Mean Fold AUC ± SD	Overall Cross-Validated AUC	Bootstrap 95% CI
Model 1	23	0.688	0.732	0.804	0.783	0.795	0.761 ± 0.049	0.757	0.642–0.861
Model 2	2	0.841	0.721	0.822	0.870	0.720	0.795 ± 0.070	0.766	0.664–0.861
Model 3	9	0.736	0.736	0.888	0.891	0.678	0.786 ± 0.098	0.786	0.696–0.868

AUC, area under the receiver operating characteristic curve; CI, confidence interval; SD, standard deviation. The same stratified fivefold partition (fixed random seed = 42) was used for all three fixed model specifications (*n* = 144; 30 HN grade 1 and 114 HN grades 5–7). Within each fold, regression coefficients were re-estimated using only the corresponding training folds and then applied to the validation fold. SNP screening, forward selection, and correlation-guided marker reduction were not repeated within folds. The overall cross-validated AUC was calculated from the combined out-of-fold prediction probabilities, whereas the mean fold AUC represents the arithmetic mean of the five fold-specific AUC values.

**Table 4:** Multivariable logistic-regression coefficients for the final 9-SNP Model 3 fitted in the training group (*n* = 86; 18 HN grade 1 and 68 HN grades 5–7).

Code	SNP ID	β	SE	*p*	OR	95% CI for OR
S01	rs12565727	0.703	0.599	0.241	2.020	0.624–6.538
S02	rs7349332	1.743	0.874	0.046	5.716	1.031–31.696
S03	rs9287638	0.689	0.486	0.156	1.991	0.768–5.161
S07	rs2073963	0.835	0.540	0.122	2.304	0.800–6.636
S10	rs9668810	−0.876	0.577	0.129	0.416	0.134–1.291
S12	rs10502861	−0.905	0.546	0.098	0.405	0.139–1.181
S18	rs1041668	−1.881	1.825	0.302	0.152	0.004–5.445
S19	rs1511061	−1.807	0.995	0.069	0.164	0.023–1.153
S25	rs16990427	0.738	1.699	0.664	2.093	0.075–58.427
Constant	—	1.200	1.234	0.331	3.319	—

Note: β values are the fitted regression coefficients. ORs and 95% CIs correspond to the target minor allele under the study coding scheme; autosomal SNPs were coded 0–2 and X-chromosomal SNPs 0/1. rs7349332 (S02) was the only predictor with *p* < 0.05 after simultaneous adjustment.

## Data Availability

The de-identified dataset generated and analyzed during the present study is not publicly available because participant consent and ethics approval did not include unrestricted public data sharing. De-identified data may be made available from the corresponding author upon reasonable request and subject to applicable ethics approval and institutional permission.

## Supplementary Materials

The following supporting information can be downloaded at: https://www.mdpi.com/article/10.3390/genes17101200/s1. Supplementary Figure S1 and Supplementary Tables S1–S7 are provided with the manuscript. The regression-coefficient table formerly presented as Supplementary Table S8 has been moved to the main text as Table 4 to facilitate interpretation and reproducibility of the final 9-SNP prediction equation. Supplementary Figure S1. Representative electropherogram images from the 23-SNP SNaPshot multiplex sensitivity assessment using 2800M control DNA (10 ng/µL stock). Serial DNA inputs of 2, 1, 0.5, 0.25, 0.125, and 0.05 ng per PCR reaction were evaluated; two replicate reactions were tested at each input. Supplementary Table S1. PCR primer sequences used for the 23-SNP multiplex panel. Supplementary Table S2. SNaPshot extension-primer sequences used for the 23-SNP multiplex panel. Supplementary Table S3. Multivariable BLRA coefficient estimates for Model 1 (23-SNP model) in the training group (n = 86). Supplementary Table S4. Historical univariable BLRA results for the 23 candidate SNPs in the training group (n = 86). The 14 markers with *p* < 0.25 were retained for further multivariable evaluation. Supplementary Table S5. Spearman correlation matrix for the five strongly intercorrelated X-chromosomal candidate markers in the training group (n = 86). Supplementary Table S6. Expected and observed SNaPshot extension-product sizes for the 23 loci using 2800M control DNA. Supplementary Table S7. Coefficient estimates and training-group performance for Model 2 (2-SNP forward conditional BLRA model).
